# Nervous Headache

**Published:** 1890-12

**Authors:** 


NERVOUS HEADACHE.—

| | | | |
|---|---|---|---|
| ℞. | Caffeine Citratis, | - - - - | grain 1. |
| | Soda Bicarb, | - - - | grains 2, |
| | Acetanilid, | - - - - - | grains 7. |

M.—Ft. pulv. No. i. Sig. At a dose.

